# Wild Seasons, Urban Stasis: Anthropogenic Food Subsidies Buffer Seasonal Dietary Shifts for Coyotes (*Canis latrans*) in a Wildland‐Urban Landscape South of Mexico City

**DOI:** 10.1002/ece3.73710

**Published:** 2026-06-08

**Authors:** Andrés Arias‐Alzate, Nitzia Flores‐Raíz, Juan F. Acevedo‐Quintero, José F. González‐Maya, Heliot Zarza

**Affiliations:** ^1^ Universidad de Antioquia Medellín Antioquia Colombia; ^2^ Independent Consultant Residencial Zacatenco México; ^3^ Grupo de investigación Ecología de Bosques Andinos Colombianos—EBAC, Escuela de Ciencias Biológicas Universidad Pedagógica y Tecnológica de Colombia—UPTC Tunja (Boyacá) Colombia; ^4^ Área Académica en Biología de la Conservación, Departamento de Ciencias Ambientales, DCBS Universidad Autónoma Metropolitana Unidad Lerma Lerma de Villada Estado de México México; ^5^ Proyecto de Conservación de Aguas y Tierras—ProCAT Colombia Bogotá Colombia

**Keywords:** *Canis latrans*, carnivore diet, dietary patterns, Louvain algorithm, prey preferences

## Abstract

Coyote ( *Canis latrans* ) populations are expanding into urban areas, yet how their feeding ecology adapts to the wildland–urban interface remains poorly understood, particularly near megacities such as Mexico City. We evaluated coyote diet across an anthropogenic gradient in the Sierra del Ajusco, comparing a conserved site (Las Rosas) and a human‐modified site (Las Maravillas). We analyzed 198 scats collected over 1 year, using prey diversity (Shannon–Wiener H′) and network modularity (Louvain algorithm) to assess spatial and seasonal variation. Mammals dominated the diet at both sites, with *Microtus mexicanus* as the primary prey. Annual dietary diversity did not differ significantly between the conserved (H′ = 2.245) and modified (H′ = 2.368) sites (*p* = 0.2502), consistent with low network modularity (0.073). In contrast, seasonal variation differed between sites: the conserved site showed a significant shift in diet diversity (*p* = 0.02587), whereas the modified site did not (*p* = 0.6874). These results indicate that the primary effect of anthropogenic disturbance is not a change in overall diet diversity, but a buffering of seasonal variation. The year‐round availability of anthropogenic resources in the modified landscape appears to decouple coyote feeding ecology from natural seasonal cycles, with potential consequences for its ecological role.

## Graphical abstract

Coyote populations are expanding into urban areas, and this study examines how their diet adapts along a gradient from conserved to human‐modified habitats. Mammals dominated the diet at both sites, and there was no significant difference in annual dietary diversity between the conserved and modified areas. Anthropogenic environments reduce seasonal variation in the coyote's diet, likely due to the constant availability of human‐related food resources.

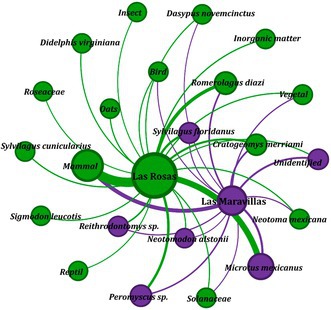

## 1 Introduction

Feeding ecology is a fundamental aspect of a species' biology and a defining feature of its ecological interactions (Christiansen and Wroe 2007). It consequently plays a critical role in population dynamics, profoundly influences the co‐evolution of interacting species, and governs underlying ecosystem processes, such as seed dispersal and predator‐prey dynamics (Christiansen and Wroe 2007; Roemer et al. 2009; Segar et al. 2020; Arias‐Alzate et al. 2022).

Mesocarnivore species (i.e., < 15 kg; Roemer et al. 2009) are vitally important in ecosystem dynamics (Penido et al. 2017; Barrera‐Vargas et al. 2023). They play a central, top‐down role by facilitating nutrient flows and regulating prey populations, either directly through predation (Penido et al. 2017; Marinho et al. 2020) or indirectly through the fear they generate (i.e., landscapes of fear; Roemer et al. 2009). Nevertheless, this group is also one of the most sensitive to environmental imbalances, particularly those driven by anthropogenic effects (Barrera‐Vargas et al. 2023). This group occupies a trophic level generally just below top predators. Some species can also act as top predators in certain socioecosystems (Roemer et al. 2009; Marinho et al. 2020) and encompasses a wide feeding spectrum, ranging from hypercarnivorous felids to omnivorous species such as certain mustelids and canids (Christiansen and Wroe 2007; Marinho et al. 2020; Arias‐Alzate et al. 2022).

Among mesocarnivores, the coyote ( *Canis latrans* ) is considered one of the most ecologically successful predators, now found virtually throughout North America (Kays et al. 2010; Bekoff and Gese 2003). This geographical expansion has been attributed to its ecological plasticity, allowing it to inhabit deforested and agricultural areas as part of its habitat (Méndez‐Carvajal and Moreno 2014; Hody and Kays 2018; Monroy‐Vilchis et al. 2020). A latitudinal pattern has been suggested in the foraging ecology of coyotes (Guerrero et al. 2004). The species is an omnivorous, opportunistic, and generalist predator that consumes everything from fruit and insects to large ungulates, carrion, and anthropogenic waste (Aranda et al. 2002; Martínez‐Vázquez et al. 2010; Hayward et al. 2023). It has been proposed that in northern Canada and the United States, coyotes feed mainly on deer, rabbits, and hares (Quinn 1997; O'Donoghue et al. 1998; Riley et al. 2003; Hayward et al. 2023), while in the southern United States, their main prey are small mammals such as rodents and rabbits (Rose and Polis 1998; Hayward et al. 2023). However, plant material was found to be the most consumed item in urban and suburban areas in Arizona (McClure et al. 1995).

In Mexico, coyotes are present throughout the entire country (Hidalgo‐Mihart et al. 2013). Within this range, their feeding ecology has been one of the most studied aspects, especially in wild populations (Aranda et al. 1995; Espinoza 2011; Cruz‐Espinoza et al. 2010; Martínez‐Vázquez et al. 2010; Ríos 2014; Uriostegui‐Velarde et al. 2015; Sedano 2019). While rodents and rabbits are often suggested as their preferred prey (Guerrero et al. 2004; Sedano 2019; Hayward et al. 2023), plant components frequently appear as the second most important food item (Espinoza 2011; Olvera 2011; Ríos 2014).

Nevertheless, significant knowledge gaps persist, particularly in the temperate forests of the Trans‐Mexican Volcanic Belt, especially in areas facing increasing urbanization and land‐use change, such as the Sierra del Ajusco, south of Mexico City (Arriola et al. 2014). Although recent studies in other fragmented regions (e.g., State of Mexico and Queretaro) also identify rodents and lagomorphs as primary prey (Olvera 2011; Ríos 2014; García et al. 2014; Espinoza‐Graciano and García‐Collazo 2017), coyote feeding ecology in wildland‐urban interfaces remains poorly understood in the country. This study evaluates coyote feeding ecology across a gradient of anthropogenic disturbance south of Mexico City by comparing the diet between a preserved pine forest and a human‐modified habitat. We also assess temporal and seasonal variations in diet within both areas. We hypothesize that the coyote diet will be more carnivorous in the conserved area and that these patterns will vary seasonally. This research provides crucial ecological data to inform management, revealing how anthropogenic pressures may fundamentally alter the temporal dynamics of coyote foraging ecology in rapidly urbanizing landscapes, such as those south of Mexico City.

## 2 Materials and Methods

### 2.1 Study Area

The study was conducted in the Sierra del Ajusco, a mountain system located in the Trans‐Mexican Volcanic Belt, within the priority terrestrial region of the Ajusco‐Chichinautzin Biological Corridor, in the border area between Mexico City and the state of Morelos (19° 09′ 03″ N, 99° 13′ 02″ W). The regional climate is classified as Cb′(w2) (semi‐cold, subhumid), with a mean annual temperature of 9.9°C and summer rainfall. Mean annual precipitation was 1524 mm during the period 2015–2025 (SMN 2026). The predominant natural vegetation is pine forest (*Pinus hartwegii*), followed by oyamel forest (*Abies religiosa*) and zacatonal grasslands (*Muhlenbergia* sp.). Currently, large areas of the region (e.g., lowlands) have been transformed by anthropogenic activities, converting the land use to agricultural and livestock purposes (Arriaga et al. 2000; Granados et al. 2004).

### 2.2 Sampling Sites

Two sampling sites were selected according to their conservation status, based on indicators such as the presence of agricultural and livestock activities. The first locality corresponds to the Las Rosas site, located within the Community Ecological Reserve “San Miguel Topilejo”, in the communal lands of San Miguel Topilejo, Mexico City (19°08′23″ N; 99°13′56″ W; 3300 m above sea level). The area is considered important for conservation due to its ecogeographic characteristics, biological diversity, and well‐preserved forest cover (Carpinteyro‐Urbán and Espinoza‐Castillo 2013). The second area corresponds to the Maravillas site, located in the communal lands of San Miguel Topilejo (19°08′ 59″ N; 99°10′52″ W; 3100 m above sea level). Land use in the area is characterized by agriculture, livestock (dominated by free‐grazing sheep), and timber extraction, with specific anthropogenic waste discharges (i.e., rubbish dumps) also present.

### 2.3 Scats Collections

At each sampling site, scats were collected opportunistically along trails and dirt roads. An approximately 8 km loop route was established and surveyed for the species' scats. These were visited at least once a month from November 2014 to November 2015. All scats were collected and identified in the field using the criteria of size, shape, color, odor, and footprints following Aranda (2012). Each scat was placed in a plastic bag labeled with the sample number, sampling site, date, and spatial location. The samples were then dried at room temperature to prevent fungal growth and were stored for diet analysis.

### 2.4 Scats Processing and Data Analysis

Each scat was processed independently to avoid bias in the analyses, following the procedure proposed by Ríos (2014). Briefly, each sample was immersed in a soapy solution for 24 h to remove fat and non‐relevant organic matter. Samples were washed under running water and manually sifted using a sieve and dissecting forceps, separating bone debris, hair, feathers, seeds, and inorganic debris. The separated items were dried at room temperature and stored in plastic bags labeled with the same information as the collection site. Subsequently, each item identified was processed and classified to the most specific taxonomic level possible.

Food items were sorted by taxonomic group as mammals, birds, reptiles, insects, and seeds. For mammals, remains (bone fragments, teeth, nails, and hair) were identified using dental guides (Aranda et al. 1995; Álvarez‐Castañeda et al. 2017) and by comparison with specimens at the Mammalogy National Collection. Other animal remains consisted of feathers and beaks (birds), limbs (insects), and scales (reptiles). Due to a high degree of degradation, these items could not be identified to the species level. Plant matter was limited to seeds, which were identified to the family level by comparison with reference material from the Seed Bank of the Faculty of Higher Studies Iztacala (UNAM).

We created a database of the consumed prey species with their respective collection and identification information: scat number, collection date, season, food category (i.e., birds, mammals, insects, reptiles, plant matter, and anthropogenic debris), spatial location, and the highest level of taxonomic identification. Subsequently, to estimate the number of scats needed for a comprehensive diet description, we performed a completeness curve analysis with 100 randomizations for both localities. This was also done by season to determine whether the collected sample size was representative (Jiménez‐Valverde and Hortal 2003). This analysis was performed using EstimateS version 9.1.0 (Colwell 2013).

Afterwards, we used the Clench Equation (Sn) to evaluate sampling quality; Sn indicates the probability of discovering new prey species as sampling effort increases (Jiménez‐Valverde and Hortal 2003). This equation (Sn = a*n/(1 + b × *n*)) considers the rate of increase of new species at the beginning of the study (*a*), a parameter related to the shape of the curve (*b*), and the sampling effort (*n*). The model was fitted using Simplex & Quasi Newton non‐linear estimation to calculate the coefficient of determination (*R* ^2^) and *a* and *b* values. Associated indices, such as slope ((*a*/1 + *b* × *n*)^2^), survey quality (Sn/a/b), and the minimum number of scats (0.95/b*(1‐0.95)) needed to record 95% of coyote prey species were also estimated. This was carried out in the software Statistica version 14.0.0.15 (TIBCO 2020).

We calculated two metrics to assess diet. First, the Percentage of Occurrence (PO) was estimated as PO = (*F*_*i*_/*N*) × 100, where *F*_*i*_ is the number of scats containing prey item *i*, and *N* is the total number of scats analyzed (Aranda et al. 2002). This metric indicates the proportion of all samples that contained a specific prey type (Ackerman et al. 1984; Martínez‐Vázquez et al. 2010). Second, to assess the contribution of each item relative to all other items found, we calculated the Relative Frequency (RF). This was calculated as RF = (*F*_*i*_/Σ*F*_*i*_) × 100, where *F*_*i*_ is the number of occurrences for item *i*, and Σ*F*_*i*_ is the total number of all prey item occurrences (the “summatory of *F*_*i*_”). This metric indicates the relative importance of each prey component within the overall diet (Ríos 2014; Martínez‐Vázquez et al. 2010).

To quantify prey diversity, we used the Shannon‐Wiener Diversity Index (H′). This index measures heterogeneity in the prey community based on the number of species and their relative abundance (Pla 2006), with low values indicating low diversity and high values indicating higher diversity (Ríos 2014). This index was used to determine differences in the annual and seasonal (i.e., dry and rainy) diets. To evaluate whether there were significant differences in annual and seasonal prey consumption at each site, we conducted a modified *t*‐test using the ecolTest package (v. 0.0.1) in R software (v. 4.0.5) (R Core Team 2021). Furthermore, we calculated maximum diversity (H′max = lnS, where ln is the natural logarithm, S is the maximum number of species), and evenness (*E* = H′/H′max). H′max represents the maximum possible diversity (assuming all species are equally present), while E measures the relationship between observed and maximum diversity. Evenness values range from 0 to 1, with values close to 0 indicating dominance by one species and values close to 1 indicating that all species are equally abundant (Magurran 1988; Soler et al. 2012).

Finally, as an alternative method for assessing dietary differences, an interaction matrix was constructed. In this matrix, rows represent the scat collection sites, and columns correspond to the identified food items. Each cell's value indicates the frequency of occurrence of an item at a given site, allowing us to assess consumption patterns relative to the level of habitat conservation. We used the Louvain algorithm (Blondel et al. 2008) to detect communities within this site‐food item interaction network. This method was chosen for its computational efficiency, its ability to optimize modularity without requiring a priori specification of community numbers, and its wide application in network ecology (Legras et al. 2019; Hervías‐Parejo et al. 2020; Hong et al. 2024). Modularity values from this algorithm range from 0 to 1. Values close to 1 indicate well‐defined communities with stronger interactions within groups than between them, while values close to 0 reflect a weak community structure with more diffuse connections and less differentiated interaction patterns.

## 3 Results

**FIGURE 1:**
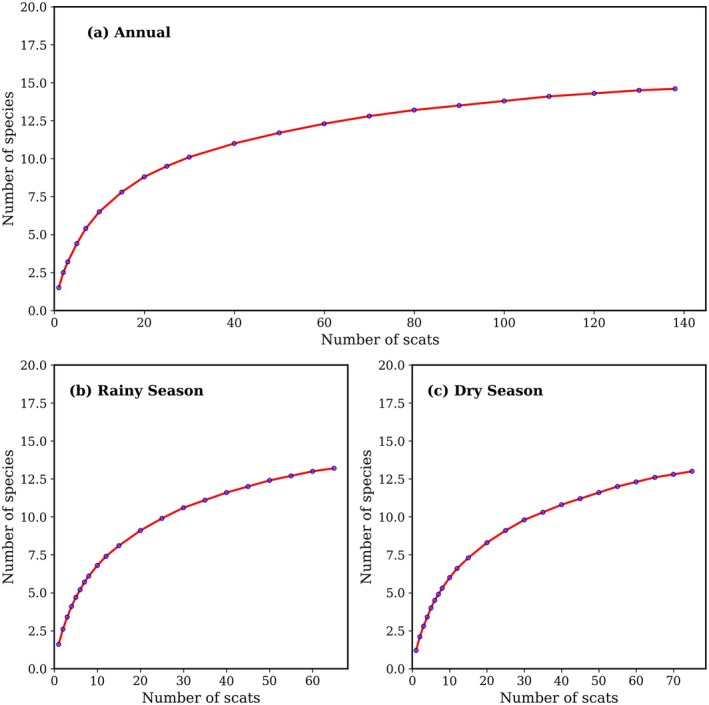
Completeness curves for number of prey species recorded in Las Rosas site (a) Annually, and during (b) Rainy and (c) Dry seasons.

**FIGURE 2:**
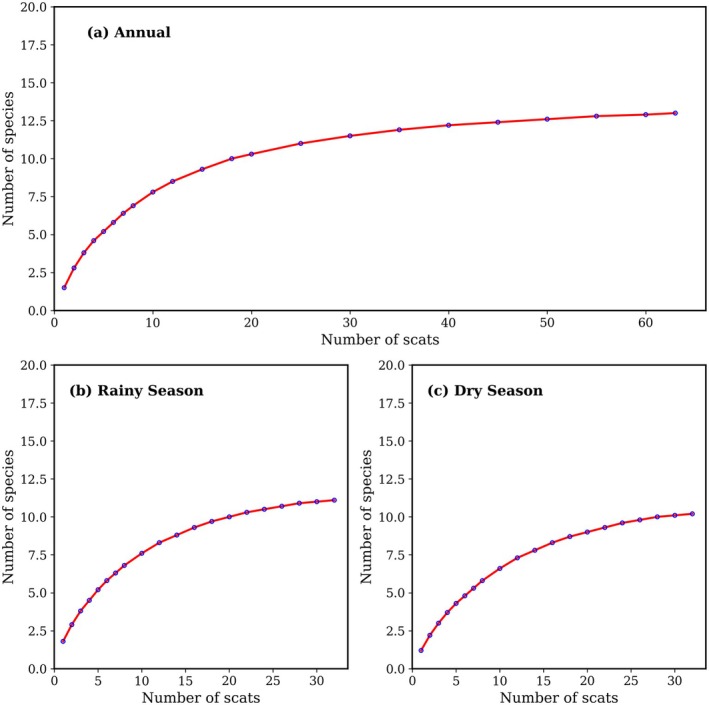
Completeness curves for number of prey species recorded in Las Maravillas site (a) Annually, and during (b) Rainy and (c) Dry seasons.

A total of 198 coyote scats was collected, with 136 samples from Las Rosas (61 rainy season, 75 dry season) and 62 from Las Maravillas (31 in each season). The completeness analysis indicates that 89% of the prey species (16 species) at the Las Rosas site were detected (*R* ^2^ = 0.996; Figure 1a). At this site, 83.5% and 87% of the prey species were detected for the rainy (*R* ^2^ = 0.999) and dry seasons, respectively (*R* ^2^ = 0.988; Figure 1b,c). Likewise, for the Las Maravillas site, this analysis indicates that 89.54% of the prey species (11 species) were detected (*R* ^2^ = 0.9985; Figure 2). This corresponded to 78.3% and 86.55% of the prey species for the rainy (*R* ^2^ = 0.999) and dry (*R* ^2^ = 0.988) seasons, respectively (Figure 2b,c). The analysis revealed that 303 and 271 scats would be required to identify 95% of the prey species in Las Rosas and Las Maravillas, respectively (Table 1).

**TABLE 1:** Representativeness of annual and seasonal coyote scat samples in the Las Rosas and Las Maravillas localities.

Site	Season	*R* ^2^	*a*	*b*	*n*	S_obs.	Slope	Inventory quality	Sampling effort
Las Rosas	Annual	0.996	1.122	0.063	136	16	0.0124	0.892	303.76
	Rainy	0.999	1.397	0.083	61	14	0.0377	0.8355	227.87
	Dry	0.988	1.115	0.07	75	14	0.0286	0.877	271.98
Las Maravillas	Annual	0.999	1.681	0.116	62	13	0.0251	0.8954	164.08
	Rainy	1	1.614	0.115	31	11	0.0775	0.7829	165.41
	Dry	0.999	2.202	0.191	31	10	0.0436	0.8655	99.68

Mammals were the most abundant category at both sites, followed by plant material and, to a lesser extent, reptiles. At the Las Rosas site specifically, 12 mammalian species were identified, belonging to four orders: Didelphimorphia, Cingulata, Rodentia, and Lagomorpha. Rodentia was the most species‐rich order, followed by Lagomorpha (Tables S1 and S2). Plant matter was identified to the family level (Poaceae, Roseaceae, and Solanaceae). Other categories (Birds, Reptiles, and Insects) could not be identified to species level due to a high degree of sample degradation.

Annually, the diet at Las Rosas was dominated by mammals (RF = 90.14%), followed by plant matter (RF = 5.16%), anthropogenic debris (RF = 1.88%), birds (RF = 1.41%), reptiles (RF = 0.94%), and insects (RF = 0.47%; Table S1). The most common prey species was *Microtus mexicanus* (PO = 51.47%), followed by *Romerolagus diazi* (PO = 25.74%; Table S3). At Las Maravillas, mammals also had the highest relative frequency (RF = 74.75%), followed by plant matter and birds (RF = 9.09% each; Table S2), and anthropogenic waste (RF = 3.03%). The most common prey species here was *Microtus mexicanus* (PO = 37.10%), followed by *Cratogeomys merriami* and *Romerolagus diazi* (PO = 20.97%; Table S4).

Seasonal variations were observed at the Las Rosas site. During the rainy season (*n* = 61 scats), mammals were the dominant category (RF = 86.87%), followed by plant matter (RF = 7.78%; including Poaceae, Solanaceae, and Roseaceae). Reptiles and anthropogenic debris were least frequent (RF = 1.11% each). At the species level, *Microtus mexicanus* had the highest percentage of occurrence (PO = 37.70%), followed by *Romerolagus diazi* (PO = 27.87%; Table S3). In the dry season (*n* = 75 scats), mammal consumption increased (RF = 92.68%), as did the occurrence of *Microtus mexicanus* (PO = 62.67%), while *Romerolagus diazi* remained stable (PO = 24%; Table S3). At the Las Maravillas site, mammals were also the most frequent category in the rainy season (*n* = 31 scats; RF = 74.75%), followed by plant matter and birds (RF = 9.09% each; Table S2). The most common prey were *Microtus mexicanus* (PO = 35.48%), *Cratogeomys merriami* (PO = 19.35%), and *Romerolagus diazi* (PO = 16.13%; Table S4). During the dry season (*n* = 31 scats), *Microtus mexicanus* (PO = 38.71%) and *Romerolagus diazi* (PO = 25.81%) remained the top prey. However, the occurrence of plant matter (PO = 22.58%), birds (PO = 16.13%), and anthropogenic debris (PO = 6.45%) increased. Reptiles and insects were not found in this season (Table S4).

Prey diversity (H′) was highest at the Las Maravillas site annually (H′=2.368 vs. 2.245) and during the dry season (H′=2.274 vs. 2.041), while Las Rosas showed higher diversity only during the rainy season (H′=2.348 vs. 2.222; Table 2). Both localities exhibited high evenness (*E* ~1), though Las Maravillas consistently demonstrated the highest values. Despite these numerical differences, a modified *t*‐test revealed no significant between‐site differences for annual (*p* = 0.2502), dry (*p* = 0.06148), or rainy (*p* = 0.3743) season diversity (Table 3). In contrast to the spatial comparison, the analysis of seasonal variation within each site revealed a striking difference. We found significant temporal variation at the Las Rosas site (*t* = 2.2445; *p* = 0.02587), but not at the Las Maravillas site (*t* = 0.4037; *p* = 0.6874).

**TABLE 2:** Shannon Diversity Index (H′), Maximum Diversity (H′max) and Evenness (E), annual and seasonal for Las Rosas and Las Maravillas localities.

Season	Las Rosas H′	H′ max	*E*	Las Maravillas H′	H′ max	*E*
Annual	2.245	2.89	0.776	2.368	2.639	0.897
Dry	2.041	2.639	0.773	2.274	2.397	0.948
Rainy	2.348	2.772	0.847	2.222	2.397	0.926

**TABLE 3:** Shannon's Diversity Index (H′), *t*‐values and Hutchinson's *t*‐test *p*‐values, annual and seasonal between Las Rosas and Las Maravillas localities.

Season	H′ Las Rosas	H′ Las Maravillas	*t*	*p*
Annual	2.244692	2.368067	1.1524	0.2502
Dry	2.040539	2.274466	1.8633	0.06148
Rainy	2.347847	2.221881	−0.89179	0.3743

**FIGURE 3:**
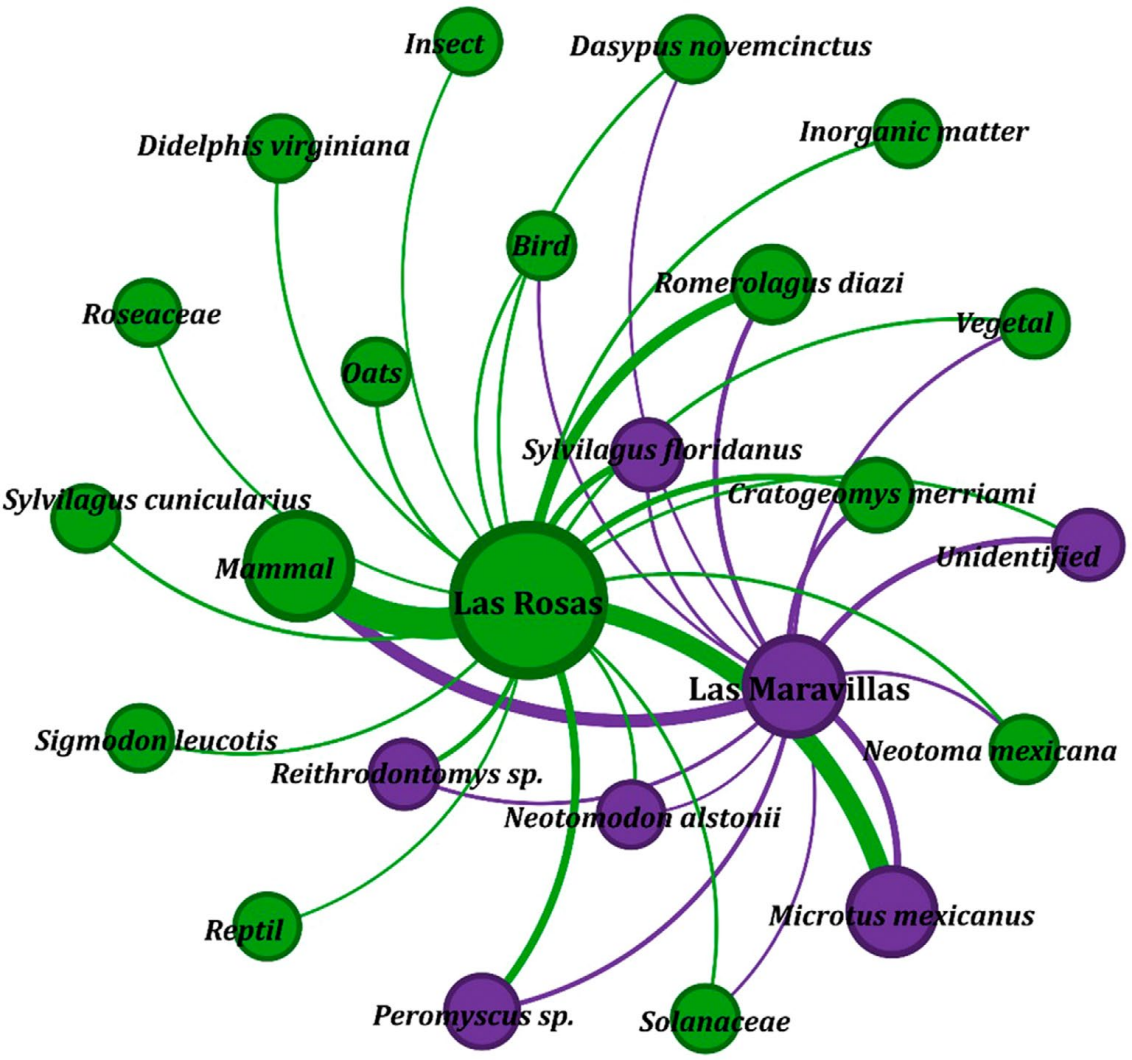
This coyote dietary interaction network examines how the species adapts along a gradient ranging from conserved habitats, “Las Rosas” (circle green), to environments modified by human activities, “Las Maravillas” (circle purple). The size of a central site node (circle) reflects the total frequency of all items at that site, while the size of a food node reflects its total frequency across the study. The thickness of the connecting lines is proportional to the frequency of occurrence of a specific item at the corresponding site.

The modularity analysis (Louvain algorithm) yielded a value of 0.073, indicating a weak community structure within the site‐food interaction network (Figure 3). This low modularity suggests there is little differentiation in the coyote diet between sites. This pattern may reflect a generalist feeding strategy or indicate that the habitat's protection level has a low influence on diet composition in this study area.

## 4 Discussion

This study highlights the ecological role of the coyote while revealing a high degree of trophic flexibility. Dietary composition did not differ markedly between the conserved (Las Rosas) and modified (Las Maravillas) sites, as indicated by non‐significant differences in diversity (*p* = 0.2502) and low network modularity (0.073). This pattern suggests that the species' generalist foraging strategy may obscure clear spatial structuring in diet. Notably, this contrasts with studies from the United States, where higher dietary diversity has been reported in highly developed environments (Rosenberg and Raphael 1986; Morey et al. 2007).

In contrast, we detected pronounced temporal variation: the conserved site exhibited significant seasonal shifts in prey diversity (*p* = 0.02587), whereas the modified site did not (*p* = 0.6874). This result suggests that anthropogenic food resources in disturbed environments may act as a stable, year‐round resource, buffering natural seasonal fluctuations in diet (Toweill and Anthony 1988; Morey et al. 2007). Such buffering likely alters trophic dynamics and may have important ecological consequences, underscoring the need for further research on its long‐term effects. Despite these patterns, our relatively large sample size (*n* = 198 scats) provides a robust basis for inference, comparable to or exceeding that of previous regional studies (Martínez‐Vázquez et al. 2010; Olvera 2011; Ríos 2014; Uriostegui‐Velarde et al. 2015; Espinoza‐Graciano and García‐Collazo 2017).

Consistent with studies across its distribution, the coyote's diet was dominated by mammals—particularly rodents and lagomorphs—which comprised the majority of prey items (Guerrero et al. 2004; Hernández 2004; Martínez‐Vázquez et al. 2010; Olvera 2011; Ríos 2014; Uriostegui‐Velarde et al. 2015; Hayward et al. 2023). This pattern matches findings from the Trans‐Mexican Volcanic Belt and the Ajusco‐Chichinautzin Biological Corridor, where mammals dominate the diet (63.15%), followed by plant matter (15.78%) (Martínez‐Vázquez et al. 2010; Olvera 2011; Ríos 2014; Uriostegui‐Velarde et al. 2015; Espinoza‐Graciano and García‐Collazo 2017). Variation in prey frequency appears to be driven primarily by local availability, reinforcing the species' feeding plasticity across conserved and human‐modified environments (Guerrero et al. 2004; Ríos 2014; Ramírez‐Albores and León‐Paniagua 2015).

This predominance of mammals likely reflects their high energetic and nutritional value, as well as their greater availability in temperate forests compared to other groups such as birds or reptiles (González 2008; Olvera 2011; Ríos 2014). Consistent with this, *Microtus mexicanus* was among the most frequently consumed species in our study, highlighting the importance of rodents in the coyote's diet (Hidalgo‐Mihart 2004; Monroy‐Vilchis 2001; Martínez‐Vázquez et al. 2010). However, this pattern varies regionally, as lagomorphs and occasionally birds can also represent major dietary components depending on local conditions (Monroy‐Vilchis 2001; Ríos 2014; Uriostegui‐Velarde et al. 2015; Espinoza‐Graciano and García‐Collazo 2017; Hayward et al. 2023).

The high representation of *Microtus mexicanus* , followed by *Romerolagus diazi* , has been widely documented in temperate forests (Sánchez 2002; Monroy et al. 2003; Hernández 2004), supporting the idea that prey consumption largely reflects local availability (Aranda et al. 1995; Hernández 2004). However, prey selection may also be shaped by traits that align with coyote foraging behavior, such as flexible activity patterns and tolerance to disturbance (Cruz‐Espinoza et al. 2010). In our study, *Peromyscus* spp. and *Cratogeomys merriami* were recorded at both localities, consistent with previous reports highlighting their importance across both conserved and human‐influenced systems (Aranda et al. 1995; Monroy‐Vilchis 2001). In particular, the high prevalence of *C. merriami* may reflect its substantial biomass contribution (~600 g) and associated energetic benefits for coyotes (Aranda et al. 1995; Bekoff and Gese 2003).

Lagomorphs represent the second most important mammalian group in the coyote diet across the Trans‐Mexican Volcanic Belt and other regions of Mexico (Ríos 2014; Uriostegui‐Velarde et al. 2015; Espinoza‐Graciano and García‐Collazo 2017; González 2008; Cruz‐Espinoza et al. 2010; Grajales‐Tam and González‐Romero 2014). In our study, *Romerolagus diazi* , *Sylvilagus floridanus* , and *Sylvilagus cunicularius* were identified, with *R. diazi* showing high representation, consistent with previous reports (Hernández 2004; Rizo‐Aguilar et al. 2016; Uriostegui‐Velarde et al. 2015). In contrast, *S. floridanus* and *S. cunicularius* were less frequently consumed despite their potential biomass contribution (Jensen et al. 2022). The higher frequency of *S. floridanus* at Las Maravillas may be related to its high reproductive rate and affinity for agricultural landscapes (Hody and Kays 2018), whereas the low occurrence of *S. cunicularius* , a species subject to hunting pressure (Gilcrease 2014), likely reflects reduced availability. Additional factors, such as interspecific interactions with other predators (e.g., *Lynx rufus* ), may also influence these patterns and warrant further evaluation (García et al. 2014; Aranda et al. 2002; Sánchez‐González et al. 2018).

The contribution of non‐mammalian groups was low, consistent with previous studies (Monroy et al. 2003; Martínez‐Vázquez et al. 2010; Olvera 2011; Ríos 2014). Plant material appears to be consumed opportunistically as a dietary supplement, although grasses may also serve purgative or anthelmintic functions (Aranda et al. 1995; Martínez‐Vázquez et al. 2010; Ríos 2014; López‐Soto et al. 2001). The presence of germinating seeds further suggests a potential role in seed dispersal (Servín 2000; Olvera 2011). Birds, reptiles, and insects were minimally represented, likely due to the high availability of mammalian prey, as these groups tend to increase in importance when mammals are scarce, particularly under anthropogenic disturbance (Aranda et al. 1995; Cruz‐Espinoza et al. 2010; Martínez‐Vázquez et al. 2010; Ríos 2014; Grajales‐Tam and González‐Romero 2014). Consistent with this pattern, reptiles were only recorded during the dry season, while insects appeared exclusively during the rainy season at Las Maravillas.

The ingestion of anthropogenic waste (e.g., plastic) appears to be incidental, likely resulting from the consumption of contaminated food resources, but it may have serious health consequences such as intestinal obstruction, perforation, or reduced intake due to false satiety (Ríos 2014; Espinoza‐Graciano and García‐Collazo 2017). Although infrequent, plastic was detected primarily in the most disturbed areas, consistent with previous observations (Olvera 2011) and patterns reported in highly urbanized systems such as southern California and Chicago (Morey et al. 2007; Parsons et al. 2022). As urban areas expand south of Mexico City, our results indicate that conserved and modified habitats can sustain similar levels of coyote dietary diversity. However, the main effect of disturbance is not spatial but temporal: the loss of seasonal variation. Coyotes in conserved areas shift their diet across seasons, whereas those in disturbed environments rely on a more constant, year‐round supply of anthropogenic resources. This buffering likely alters trophic dynamics and the species' ecological role, highlighting that even highly adaptable mesocarnivores are functionally affected by human‐induced change.

## Author Contributions

**Andrés Arias‐Alzate:** data curation (equal), formal analysis (equal), methodology (equal), visualization (equal), writing – original draft (lead), writing – review and editing (equal). **Nitzia Flores‐Raíz:** data curation (equal), formal analysis (equal), investigation (equal), methodology (equal), writing – review and editing (supporting). **Juan F. Acevedo‐Quintero:** data curation (equal), formal analysis (equal), software (lead), writing – review and editing (equal). **José F. González‐Maya:** data curation (equal), formal analysis (equal), software (equal), validation (equal), visualization (equal), writing – review and editing (lead). **Heliot Zarza:** conceptualization (lead), funding acquisition (lead), investigation (equal), methodology (equal), project administration (lead), supervision (equal), writing – review and editing (equal).

## Funding

This work was supported by Universidad Autónoma Metropolitana, 54302011.

## Conflicts of Interest

The authors declare no conflicts of interest.

## Supporting information


**Table S1.** Total and seasonal relative frequency (RF) of prey‐species found in the scats at Las Rosas site.
**Table S2.** Total and seasonal relative frequency (RF) of prey‐species found in the scats at Las Maravillas site.
**Table S3.** Total and seasonal percentage of occurrence (PO) of prey‐species found in the scats at Las Rosas site.
**Table S4.** Total and seasonal percentage of occurrence (PO) of prey‐species found in the scats at Las Maravillas site.

## Data Availability

All the required data are uploaded as Supporting Information. Also, the raw data for this study are available in the Zenodo repository: https://doi.org/10.5281/zenodo.19837458 (Zarza et al. 2026).
