# Supplementary material for: Wild Seasons, Urban Stasis: Anthropogenic Food Subsidies Buffer Seasonal Dietary Shifts for Coyotes (Canis latrans) in a Wildland‐Urban Landscape South of Mexico City
Source: Ecol Evol. 2026 Jun 8;16(6):e73710. doi: 10.1002/ece3.73710 (PMC13244077; doi:10.1002/ece3.73710)
Supplement: Supplementary file 1 — Table S1. Total and seasonal relative frequency (RF) of prey‐species found in the scats at Las Rosas site. Table S2. Total and seasonal relative frequency (RF) of prey‐species found in the scats at Las Maravillas site. Table S3. Total and seasonal percentage of occurrence (PO) of prey‐species found in the scats at Las Rosas site. Table S4. Total and seasonal percentage of occurrence (PO) of prey‐species found in the scats at Las Maravillas site. [file ECE3-16-e73710-s001.docx]

**Supplemental material**

**Table S1. Total and seasonal relative frequency (RF) of prey-species found in the scats at Las Rosas site**.

| **Category** | **Family** | **Species** | **RF Annual** | **RF Dry** | **RF Rainy** |
| --- | --- | --- | --- | --- | --- |
| **MAMMALS (ORDEN)** |  |  | **90.14** | **92.68** | **86.67** |
| Didelphimorphia | Didelphidae | *Didelphis virginiana* | 1.41 | 0.0 | 3.33 |
| Cingulata | Dasypodidae | *Dasypus novemcinctus* | 1.41 | 0.81 | 2.22 |
| Rodentia | Geomyidae | *Cratogeomys merriami* | 7.51 | 8.94 | 5.56 |
|  | Cricetidae | *Microtus mexicanus* | 32.86 | 38.21 | 25.56 |
|  |  | *Neotoma mexicana* | 1.88 | 0.81 | 3.33 |
|  |  | *Neotoma alstoni* | 1.41 | 0.81 | 2.22 |
|  |  | *Peromyscus* sp*.* | 10.33 | 10.57 | 10.00 |
|  |  | *Reithrodontomys* sp. | 4.23 | 4.88 | 3.33 |
|  |  | *Sigmodon leucotis* | 1.41 | 2.44 | 0.0 |
| Lagomorpha | Leporidae | *Romerolagus diazi* | 16.43 | 14.63 | 18.89 |
|  |  | *Sylvilagus cunicularius* | 2.35 | 3.25 | 1.11 |
|  |  | *Sylvilagus floridanus* | 8.92 | 7.32 | 11.11 |
| **REPTILES** |  |  | 0.94 | 0.81 | 1.11 |
| **BIRDS** |  |  | 1.41 | 0.0 | 3.33 |
| **INSECTS** |  |  | 0.47 | 0.81 | 0.0 |
| **PLANT MATTER** |  |  | 5.16 | 3.25 | 7.78 |
| *Poales* |  | Poaceae | 3.29 | 3.25 | 3.33 |
| *Rosales* |  | Rosaceae | 0.47 | 0.0 | 1.11 |
| *Solanales* |  | Solanaceae | 1.41 | 0.0 | 3.33 |
| **ANTHROPOGENIC DEBRIS** |  |  | 1.88 | 2.44 | 1.11 |

**Table S2. Total and seasonal relative frequency (RF) of prey-species found in the scats at Las Maravillas site.**

| **Category** | **Family** | **Species** | **RF Annual** | **RF Dry** | **RF Rainy** |
| --- | --- | --- | --- | --- | --- |
| **MAMMALS (ORDEN)** |  |  | **74.75** | **74.55** | **75.00** |
| Didelphimorphia | Didelphidae | *Didelphis virginiana* | 0.00 | 0.00 | 0.00 |
| Cingulata | Dasypodidae | *Dasypus novemcinctus* | 1.01 | 0.00 | 2.27 |
| Rodentia | Geomyidae | *Cratogeomys merriami* | 13.13 | 12.73 | 13.64 |
|  | Cricetidae | *Microtus mexicanus* | 23.23 | 21.82 | 25.00 |
|  |  | *Neotoma mexicana* | 4.04 | 7.27 | 0.00 |
|  |  | *Neotoma alstoni* | 2.02 | 3.64 | 0.00 |
|  |  | *Peromyscus* sp*.* | 9.09 | 9.09 | 9.09 |
|  |  | *Reithrodontomys* sp*.* | 5.05 | 1.82 | 9.09 |
|  |  | *Sigmodon leucotis* | 1.01 | 0.00 | 2.27 |
|  |  | *Romerolagus diazi* | 13.13 | 14.55 | 11.36 |
| Lagomorpha | Leporidae | *Sylvilagus cunicularius* | 0.00 | 0.00 | 0.00 |
|  |  | *Sylvilagus floridanus* | 3.03 | 3.64 | 2.27 |
| **REPTILES** |  |  | 0.00 | 0.00 | 0.00 |
| **BIRDS** |  |  | 9.09 | 9.09 | 9.09 |
| **INSECTS** |  |  | 4.04 | 0.00 | 9.09 |
| **PLANT MATTER** |  |  | 9.09 | 12.73 | 4.55 |
| *Poales* | Poaceae |  | 7.07 | 9.09 | 4.55 |
| *Rosales* | Rosaceae |  | 0.00 | 0.00 | 0.00 |
| *Solanales* | Solanaceae |  | 2.02 | 3.64 | 0.00 |
| **ANTHROPOGENIC DEBRIS** |  |  | 3.03 | 3.64 | 2.27 |

**Table S3. Total and seasonal percentage of occurrence (PO) of prey-species found in the scats at Las Rosas site**.

| **Category** | **Family** | **Species** | **PO Annual** | **PO Dry** | **PO Rainy** |
| --- | --- | --- | --- | --- | --- |
| **MAMMALS (ORDEN)** |  |  | **141.18** | **152.00** | **127.87** |
| Didelphimorphia | Didelphidae | *Didelphis virginiana* | 2.21 | 0.00 | 4.92 |
| Cingulata | Dasypodidae | *Dasypus novemcinctus* | 2.21 | 1.33 | 3.28 |
| Rodentia | Geomyidae | *Cratogeomys merriami* | 11.76 | 14.67 | 8.20 |
|  | Cricetidae | *Microtus mexicanus* | 51.47 | 62.67 | 37.70 |
|  |  | *Neotoma mexicana* | 2.94 | 1.33 | 4.92 |
|  |  | *Neotoma alstoni* | 2.21 | 1.33 | 3.28 |
|  |  | *Peromyscus* sp*.* | 16.18 | 17.33 | 14.75 |
|  |  | *Reithrodontomys* sp. | 6.62 | 8.00 | 4.92 |
|  |  | *Sigmodon leucotis* | 2.21 | 4.00 | 0.00 |
| Lagomorpha |  | *Romerolagus diazi* | 25.74 | 24.00 | 27.87 |
|  | Leporidae | *Sylvilagus cunicularius* | 3.68 | 5.33 | 1.64 |
|  |  | *Sylvilagus floridanus* | 13.97 | 12.00 | 16.39 |
| **REPTILES** |  |  | 1.47 | 1.33 | 1.64 |
| **BIRDS** |  |  | 2.21 | 0.00 | 4.92 |
| **INSECTS** |  |  | 0.74 | 1.33 | 0.00 |
| **PLANT MATTER** |  |  | 8.09 | 5.33 | 11.48 |
| *Poales* |  | Poaceae | 5.15 | 5.33 | 4.92 |
| *Rosales* |  | Rosaceae | 0.74 | 0.00 | 1.64 |
| *Solanales* |  | Solanaceae | 2.21 | 0.00 | 4.92 |
| **ANTHROPOGENIC DEBRIS** |  |  | 2.94 | 4.00 | 1.64 |

**Table S4. Total and seasonal percentage of occurrence (PO) of prey-species found in the scats at Las Maravillas site.**

| **Category** | **Family** | **Species** | **PO Annual** | **PO Dry** | **PO Rainy** |
| --- | --- | --- | --- | --- | --- |
| **MAMMALS (ORDEN)** |  |  | **119.35** | **132.26** | **106.45** |
| Didelphimorphia | Didelphidae | *Didelphis virginiana* | 0.00 | 0.00 | 0.00 |
| Cingulata | Dasypodidae | *Dasypus novemcinctus* | 1.61 | 0.00 | 3.23 |
| Rodentia | Geomyidae | *Cratogeomys merriami* | 20.97 | 22.58 | 19.35 |
|  | Cricetidae | *Microtus mexicanus* | 37.10 | 38.71 | 35.48 |
|  |  | *Neotoma mexicana* | 6.45 | 12.90 | 0.00 |
|  |  | *Neotoma alstoni* | 3.23 | 6.45 | 0.00 |
|  |  | *Peromyscus* sp*.* | 14.52 | 16.13 | 12.90 |
|  |  | *Reithrodontomys* sp*.* | 8.06 | 3.23 | 12.90 |
|  |  | *Sigmodon leucotis* | 1.61 | 0.00 | 3.23 |
|  |  | *Romerolagus diazi* | 20.97 | 25.81 | 16.13 |
| Lagomorpha | Leporidae | *Sylvilagus cunicularius* | 0.00 | 0.00 | 0.00 |
|  |  | *Sylvilagus floridanus* | 4.84 | 6.45 | 3.23 |
| **REPTILES** |  |  | 0.00 | 0.00 | 0.00 |
| **BIRDS** |  |  | 14.52 | 16.13 | 12.90 |
| **INSECTS** |  |  | 6.45 | 0.00 | 12.90 |
| **PLANT MATTER** |  |  | 14.52 | 22.58 | 6.45 |
| *Poales* | Poaceae |  | 11.29 | 16.13 | 6.45 |
| *Rosales* | Rosaceae |  | 0.00 | 0.00 | 0.00 |
| *Solanales* | Solanaceae |  | 3.23 | 6.45 | 0.00 |
| **ANTHROPOGENIC DEBRIS** |  |  | 4.84 | 6.45 | 3.23 |
